# IL-36γ Is a Strong Inducer of IL-23 in Psoriatic Cells and Activates Angiogenesis

**DOI:** 10.3389/fimmu.2018.00200

**Published:** 2018-02-26

**Authors:** Charlie Bridgewood, Gareth W. Fearnley, Anna Berekmeri, Philip Laws, Tom Macleod, Sreenivasan Ponnambalam, Martin Stacey, Anne Graham, Miriam Wittmann

**Affiliations:** ^1^Centre of Skin Sciences, School of Chemistry and Biosciences, University of Bradford, Bradford, United Kingdom; ^2^Endothelial Cell Biology Unit, School of Molecular and Cellular Biology, University of Leeds, Leeds, United Kingdom; ^3^Department of Dermatology, Chapel Allerton Hospital, Leeds, United Kingdom; ^4^Faculty of Biological Sciences, School of Molecular and Cellular Biology, University of Leeds, Leeds, United Kingdom; ^5^National Institute of Health Research (NIHR), Leeds Biomedical Research Centre (BRC), Chapel Allerton Hospital, Leeds, United Kingdom; ^6^Biomedical Sciences, School of Chemistry and Biosciences, University of Bradford, Bradford, United Kingdom; ^7^Leeds Institute of Rheumatic and Musculoskeletal Medicine (LIRMM), University of Leeds, Leeds, United Kingdom

**Keywords:** psoriasis, IL-36γ, IL-23, macrophages, monocytes, angiogenesis, endothelial, inflammation

## Abstract

The IL-1 family member cytokine IL-36γ is recognised as key mediator in the immunopathology of psoriasis, hallmarks of which involve the activation of both resident and infiltrating inflammatory myeloid cells and aberrant angiogenesis. This research demonstrates a role for IL-36γ in both myeloid activation and angiogenesis. We show that IL-36γ induces the production of psoriasis-associated cytokines from macrophages (IL-23 and TNFα) and that this response is enhanced in macrophages from psoriasis patients. This effect is specific for IL-36γ and could not be mimicked by other IL-1 family cytokines such as IL-1α. IL-36γ was also demonstrated to induce endothelial tube formation and branching, in a VEGF-A-dependent manner. Furthermore, IL-36γ-stimulated macrophages potently activated endothelial cells and led to increased adherence of monocytes, effects that were markedly more pronounced for psoriatic macrophages. Interestingly, regardless of stimulus, psoriasis monocytes showed increased adherence to both the stimulated and unstimulated endothelium when compared with monocytes from healthy individuals. Collectively, these findings show that IL-36γ has the potential to enhance endothelium directed leucocyte infiltration into the skin and strengthen the IL-23/IL-17 pathway adding to the growing evidence of pathogenetic roles for IL-36γ in psoriatic responses. Our findings also point to a cellular response, which could potentially explain cardiovascular comorbidities in psoriasis in the form of endothelial activation and increased monocyte adherence.

## Introduction

Psoriasis is an immune mediated inflammatory disease which affects 2–3% of the world’s population (1). Psoriatic lesions manifest as hyperkeratotic plaques, dermo-epidermal inflammation, and aberrant blood vessel formation caused by the complex interplay between tissue resident cells, dendritic cells, macrophages, and T cells and resultant enhanced expression of the IL-23–Th17/Th22 and IL-12–IFNγ/TNFα pathways (2).

IL-36α, IL-36β, and IL-36γ are members of the IL-1 family of cytokines that signal through a common receptor composed of IL-36 receptor (IL-36R) and IL-1R/AcP to activate NF-κB and MAPKs, such as p38 and JNK, and promote inflammatory responses.

IL-36α, IL-36β, and IL-36γ are members of the wider IL-1 family of cytokines. These cytokines mediate inflammatory events through the IL-36R and activate NF-κB and MAPKs, such as p38 and JNK in susceptible cells. The significance of IL-36γ for psoriatic inflammation is increasingly being recognised (3–6). IL-36 cytokines, in particular IL-36γ is dramatically upregulated in lesional psoriasis when compared with healthy controls (5). As well as acting as a psoriatic biomarker, loss-of-function mutations in the IL-36R antagonist (IL-36RA) in multiple cohorts of generalised pustular psoriasis (GPP) patients provide evidence that IL-36 plays a causative role in the pathology of psoriasis (7–9). IL-36 has recently also been implicated in other skin inflammatory diseases including acne and hidradenitis suppurativa, and allergic contact dermatitis (10, 11). IL-36γ, which is highly expressed by epithelial cells, is thought to be released in the context of cell damage or *via* non-conventional secretory pathways (12–14). Following release, it has been shown that IL-36γ is processed into its bioactive form by cathepsin S and results in the subsequent stimulation of surrounding tissues (15). IL-36R-mediated signal transduction has been shown to induce the release of pro-inflammatory cytokines (e.g., IL-8, TNFα, and IL-6), upregulate antimicrobial peptides and proliferative mediators such as defensins and HB-EGF, as well as T cell attracting or polarising cytokines such as CCL20 and IL-12, respectively (16–19).

Angiogenesis is the formation of new blood vessels from the preexisting vasculature and is a hallmark of psoriasis lesions (20). Microvascular changes within psoriasis lesions include pronounced dilation, increased permeability and endothelial cell proliferation. Immature permeable blood vessels may enhance dermal inflammation through immune cell recruitment (21, 22). A recent study confirmed a positive correlation between hypervascularisation and disease severity (23). Excessive capillary-venular dilatation precedes development of psoriatic inflammation, and resolution of these vascular changes is associated with remission of psoriasis lesions (24). VEGF-A is thought to be the driving force behind angiogenesis observed in psoriatic lesions. Mice that overexpress VEGF-A show an inflammatory response that histologically resembles psoriasis (25, 26). The *VEGFA* gene is located on chromosome 6 at 6p21, close to PSORS 1, which is a known chromosomal locus for psoriasis susceptibility (27, 28). The +405 CC *VEGFA* genotype, also known as the “high VEGF-A-producing genotype,” is associated with early onset psoriasis, whereas the “low VEGF-A-producing genotype” has no association with psoriasis (29–31). This suggests that the pro-angiogenic potential of an individual may influence disease progression.

Treatment of human psoriasis with biologics has unequivocally shown that activation of the IL-23/IL-17 pathway is key for clinical symptom development (32). IL-23 induces and maintains the differentiation of IL-17- and IL-22-producing lymphocytes, which serve as the primary source of IL-17 and IL-22, both of which orchestrate epidermal hyperplasia and tissue inflammation in psoriasis (2). In murine induced psoriasis models, infiltrating macrophages, monocytes, and monocyte-derived dendritic cells and their subsequent T cell activating cytokines such as IL-23 have been shown to drive inflammation (33–37). A mechanistic link between IL-36 and the IL-23/IL-17 axis is becoming increasingly clear (6, 38–40). Work on other inflammatory skin diseases has also highlighted a correlation between IL-36 and IL-17 (41, 42).

Whilst previous reports have shown that IL-36γ induces inflammatory mediators from macrophages, little is known about its ability to induce psoriasis relevant cytokines such as TNFα and IL-23 (16). The ability of IL-36γ to induce such inflammatory mediators from infiltrating macrophages could escalate the inflammatory cascade by activating surrounding fibroblasts, endothelial cells (18), and keratinocytes and ultimately lead to further immune cell recruitment. In recent studies, GPP patients with DITRA (Deficiency of IL-36R Antagonist) showed significant disease improvement after receiving monocyte apheresis therapy, highlighting the potential importance of an IL-36-macrophage axis in the pathology of psoriasis (43, 44).

In this study, we highlight the role of IL-36γ in both macrophage and vascular activation in the context of psoriatic lesions. Our data demonstrate that IL-36γ induces the secretion of a key driver of psoriasis, IL-23, by macrophages and that this induction is enhanced in macrophages of psoriasis patients. IL-36γ also induces angiogenesis and branching of endothelial cells in a VEGF-A-dependent manner. Supernatant from IL-36γ treated macrophages potently activate endothelial cells and increased ICAM-1 expression. Psoriasis monocytes show an increased adhesion to both stimulated and untreated endothelial cells. Overall, the presented findings add to the growing body of evidence for IL-36γ as highly relevant molecule in psoriasis immunopathology.

## Materials and Methods

### Cell Isolations and Cell Culture

Blood was collected in sodium citrate tubes. PBMCs were separated using Lymphoprep density gradient centrifugation. Monocytes were isolated from PBMCs using magnetic separation CD14+ beads (Miltenyi Biotech) using the Dynal MPC column (Invitrogen, CA, USA). Monocytes were resuspended in RPMI (ThermoFisher Scientific, MA, USA) containing 10% FCS and penicillin/streptomycin (100 U/100 mg/ml; both Life Technologies, Carlsbad, CA, USA). CD14+ purity was tested by FACs analysis with mouse antihuman CD14 FITC conjugated or mouse IgG isotype control (both 1:100; both ImmunoTools, Friesoythe, Germany). Purity for healthy patients was >90% (Figure S2 in Supplementary Material). Umbilical cords were supplied by Bradford Royal Infirmary under the approval and processing of Ethical Tissue Bradford. Human umbilical vein endothelial cells (HUVECs) were isolated from umbilical cords in a previously described method (45). Monocytes were seeded onto plates (dependent on application) in RPMI overnight to generate day 1 macrophages.

### Macrophage Purity and IL-36R Confirmation

Isolated macrophages were seeded onto coverslips overnight. Cells were washed in PBS and fixed in 4% formaldehyde for 20 min. Cells were then blocked for 1 h in 5% BSA in PBS. Cells were incubated overnight with rabbit anti-human IL-36R 1:500 (Novus Biologics, Littleton, CO, USA) or rabbit IgG isotype control (1:500; Abcam, Cambridge, UK). Cells were then washed with PBS and incubated with donkey anti-rabbit Alexa 594 conjugated and mouse anti-human CD14 FITC conjugated or mouse IgG isotype control (both 1:100; both ImmunoTools, Friesoythe, Germany). Cells were visualised using the EVOS XL microscope (Thermo Fisher Scientific).

### Patient Demographics

Details on patients who gave blood for the study are listed below in (Table 1). All patients included are under care in the dermatology department and have a diagnosis of plaque psoriasis; one patient presented mainly with palmoplantar pustular psoriasis at the time point blood was taken. Patients receiving conventional systemic treatment known to change the biological response of leucocytes, in particular methotrexate, cyclosporine A, or leflunomide were excluded from the study. For this experimental setup, where cells were isolated involving multiple washing steps, cell culture and *ex vivo* stimulation, biologics treatment was not an exclusion criteria. We carefully checked the dataset, and there was no tendency for a difference in our outcome measured between cells derived from patients with or without biologics treatment.

**Table 1:** Patient demographics.

Diagnosis	Age	Gender	Disease duration (years)	PsA	PASI	Current systemic treatment
Plaque psoriasis	48	Female	42	Yes	Minimal disease activity	Secukinumab
Plaque psoriasis	38	Male	10	No	14.4	None
Plaque psoriasis	43	Female	Unknown	No	Minimal disease activity	Ustekinumab
Plaque psoriasis	55	Male	10	No	Minimal disease activity	None
Plaque psoriasis	41	Male	10	Yes	Minimal disease activity	Adalimumab
Plaque psoriasis	24	Female	13	No	5.1	None
Plaque psoriasis	52	Male	13	No	6	Ustekinumab
Plaque psoriasis	48	Male	10	No	8.1	None
Plaque psoriasis	29	Female	22	No	7.7	None
Plaque psoriasis	41	Female	28	Yes	1.2	Ustekinumab
Plaque psoriasis	35	Male	15	No	4	Ustekinumab
Plaque psoriasis	37	Male	10	No	8	None
Plaque psoriasis	48	Female	30	Yes	13	None
Pustular palmoplantar and plaque psoriasis	70	Female	Unknown	No	Minimal disease activity	Ustekinumab

*PsA, psoriatic arthritis; PASI, Psoriasis Area and Severity Index*.

As for the healthy controls, none were known to suffer from psoriasis, eczema or any active inflammatory disease under systemic treatment. Healthy controls were matched regarding gender distribution; the age range was between 28 and 52.

### Macrophage Cytokine Stimulation

Monocytes were seeded at 1 × 10^5^ in 96-well plates (Greiner Bio-One, Stonehouse, UK) in RPMI overnight to generate day 1 macrophages. Where relevant, macrophages were primed for 24 h with IFNγ 20 ng/ml. Macrophages were stimulated with IL-36γ protein, which was generated as previously described (15, 46), IL-17, TNFα, and IL-1α (PeproTech, Rocky Hill, NJ, USA). Following 48 h stimulation, supernatant was stored at −80°C. Concentrations of IL-23 and TNFα were measured using ELISA kits from eBioscience/ThermoFisher (Waltham, MA, USA). ELISAs were carried out according to the manufacturer’s protocols. Reproducibility of the supernatants was confirmed by triplicate testing, with <10% error.

### Tubulogenesis Assay

Primary human foreskin fibroblasts (PromoCell, Heidelberg, Germany) were cultured in 48-well plates in complete DMEM [containing 10% (v/v) FCS, 1% (v/v) non-essential amino acids and 1% (v/v) sodium pyruvate] until confluent. 6,500 HUVECs were seeded onto the fibroblasts monolayer in a 1 ml 1:1 mixture of complete DMEM and ECGM (PromoCell). Cells were left to acclimatise for 24 h. Media were aspirated and replaced with fresh ECGM ± growth factors (VEGF-A, 10 ng/ml) or IL-36 (50 ng/ml) or inhibitors (IL-36RA, 50 ng/ml), Sutent (Sigma, 1 nM) or anti-VEGF-A neutralising antibody (R&D Systems, 50 or 100 ng/ml) as indicated; media were replaced every 2–3 days for 9 days. Cocultures were fixed in 200 µl 10% (v/v) formalin for 20 min and blocked in 5% (w/v) BSA for 30 min at RT. Cocultures were then incubated with 1 µg/ml mouse anti-human PECAM-1 (CD31) (Santa Cruz, Dallas, TX, USA) overnight at 4°C. Cells were washed three times with PBS before incubation with anti-mouse Alexa Fluor 594 conjugate (Invitrogen) for 3 h at RT. Wells were washed three times with PBS. Endothelial tubules were visualised *via* immunofluorescence microscopy using an EVOS-fl inverted digital microscope (Thermo Fisher Scientific). Three random fields were imaged per well. Total tubule length and number of branch points were quantified from each photographic field using the open source software AngioQuant (www.cs.tut.fi/sgn/csb/angioquant) and values were averaged. For a more detailed method, see Ref. (47).

### VEGF-A Induction Quantification by Immunoblot and ELISA

Endothelial or fibroblast cells were seeded into 6-well plates and cultured in ECGM or complete DMEM until ~80% confluent. Cells were then washed twice with PBS and starved in MCDB131 + 0.2% (w/v) BSA for 2 h before stimulation with IL-36 (50 ng/ml) for 24 h. Cells were then washed twice with ice-cold PBS and lysed in 2% (w/v) SDS, TBS, 1 mM PMSF and protease inhibitor cocktail (Sigma-Aldrich). Protein concentration was determined using the bicinchoninic acid assay (ThermoFisher). 20 µg of protein lysate was subjected to SDS-PAGE before transfer onto nitrocellulose membrane and analysis *via* immunoblotting using antibodies against VEGF-A, VEGFR1, and VEGFR2 (R&D Systems). For a detailed immunoblot protocol, see Ref. (48). The relative expression of the non-stimulated control was set to 1, and all other results expressed as a ratio of this. To measure VEGF-A secretion from both cell types, the supernatant was tested using VEGF-A ELISA kit (eBioscience/ThermoFisher).

### Macrophage Supernatant-Endothelial Activation Assay

Following 48 h stimulation, supernatant was removed and stored at −80°C. HUVEC was cultured on black TC grade fluorescence plates (PerkinElmer, Waltham, MA, USA), in PromoCell endothelial cell media containing penicillin/streptomycin (100 U/100 mg/ml) (Life Technologies, Carlsbad, CA, USA). Supernatant was cultured with HUVEC at ratio of 1:10 for 24 h. Recombinant IL-36γ was added to control wells to serve as a blank. After 24 h, the cells were fixed for 15 min with 4% formaldehyde in PBS. Mouse anti-human ICAM-1 FITC or mouse IgG isotype control was added (1:500) (BioLegend, San Diego, CA, USA). The fluorescence intensity of each well was measured using the Promega GloMax plate reader (Madison, WI, USA). For immunocytochemistry, the cells were visualised using the EVOS XL microscope.

### Monocyte Adherence Assays

Monolayers of HUVEC were grown to confluence in 24-well plates (Greiner Bio-One). HUVECs were stimulated with macrophage supernatant as above, or with TNFα (10 ng/ml) for 24 h and then suspended in fresh media before experiments. 1 × 10^5^ monocytes were added per chamber for 30 min. After 30 min, non-adherent cells were washed away, and the cells were fixed using 4% formaldehyde in PBS and blocked in 5% BSA in PBS for 1 h. The cells were the stained with mouse anti-human CD14 FITC conjugated or mouse IgG isotype control (both 1:100) (both ImmunoTools). The cells were visualised using the EVOS XL microscope, and the number of adhered cells counted using ImageJ software.

### Statistical Analysis

This was performed using a one-way analysis of variance (ANOVA) followed by Tukey’s *post hoc* test, two-way ANOVA followed by Bonferroni multiple comparison or single unpaired *t*-test using GraphPad Prism software (La Jolla, CA, USA). Significant differences between control and test groups were evaluated with *p* values less than *p < 0.05, **p < 0.01, ***p < 0.001, and ****p < 0.0001 indicated on the graphs. Error bars represent the SEM.

## Results

### IL-36γ Induces Increased IL-23 and TNFα from Psoriasis Macrophages

Psoriasis is driven by aberrant type-3 immune responses, characterised by high levels of IL-17 and IL-22 (32, 49). A key inducer of type-3 responses is IL-23, which is expressed by antigen-presenting cells including macrophages upon stimulation with TLRs agonists such as LPS and flagellin (50, 51). IL-36γ is an abundant and prominent mediator in skin psoriasis, and we were interested in its ability to induce IL-23 expression by macrophages.

**Figure 1:**
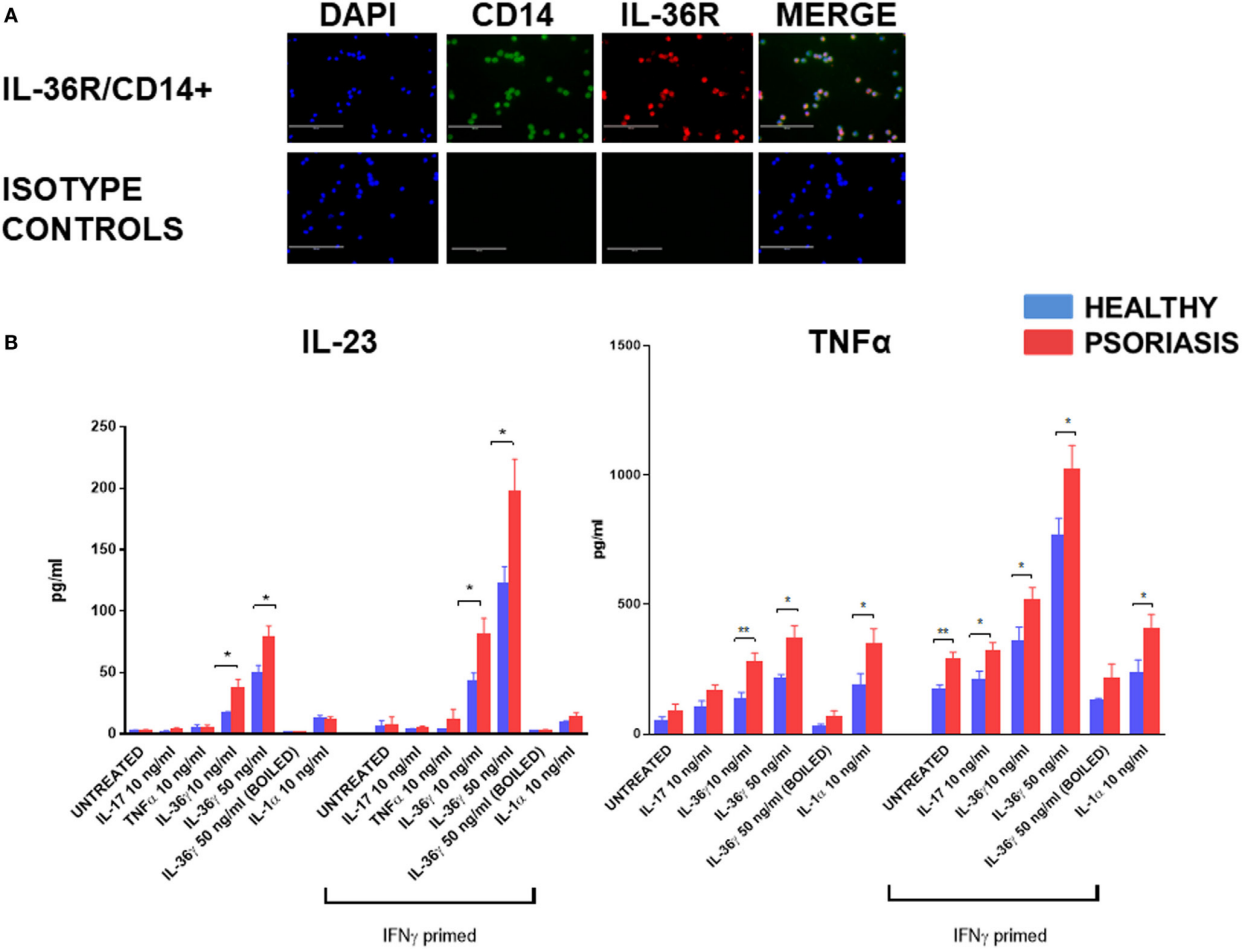
**(A)** IL-36 receptor (IL-36R) (red) on macrophages; CD14+ (green) (magnification 20×). **(B)** Following 48 h stimulation with IL-36γ, IL-1α, IL-17A, TNFα, and IFNγ (24 h priming), supernatant was analysed by ELISA for TNFα and IL-23. Unpaired *t*-test *p < 0.05, **p < 0.01 psoriasis versus healthy (sample size: psoriasis = 9, healthy = 9, and boiled control *n* = 3).

In support of previous mRNA data (52) IL-36R protein was found to be expressed by blood derived CD14+ monocyte/macrophages (Figure 1A) (*n* = 3, healthy). To assess the functional significance of IL-36γ interactions with macrophages, IL-36γ stimulation was performed for 48 h before analysis of TNFα and IL-23 secretion *via* ELISA (Figure 1B). As macrophages are known to be sensitive to LPS stimulation, boiled IL-36γ was included as a control for potential endotoxin contamination of the protein preparation. TNFα induction was measured at 24 and 48 h (Figure S1 in Supplementary Material). Differences between treatment groups became more apparent when more time was allowed for the secreted mediator to accumulate. Both 10 and 50 ng/ml IL-36γ induced a significant increase in IL-23 secretion when compared with unstimulated cells, which was further amplified when the macrophages were primed with IFNγ 20 ng/ml. For both doses of IL-36γ, macrophages from psoriatic donors secreted significantly more IL-23 compared with cells from healthy individuals. Other psoriasis relevant mediators such as IL-17, TNFα, and IL-1 did not induce a significant increase in IL-23 secretion, regardless of IFNγ priming. IL-36γ also induced significant TNFα secretion from macrophages, as did both IL-1 and IL-17 when compared with untreated controls. However, following IFNγ priming, IL-36γ induced secretion exceeded both IL-1 and IL-17.

### IL-36-Stimulated Endothelial Cell Tubulogenesis

**Figure 2:**
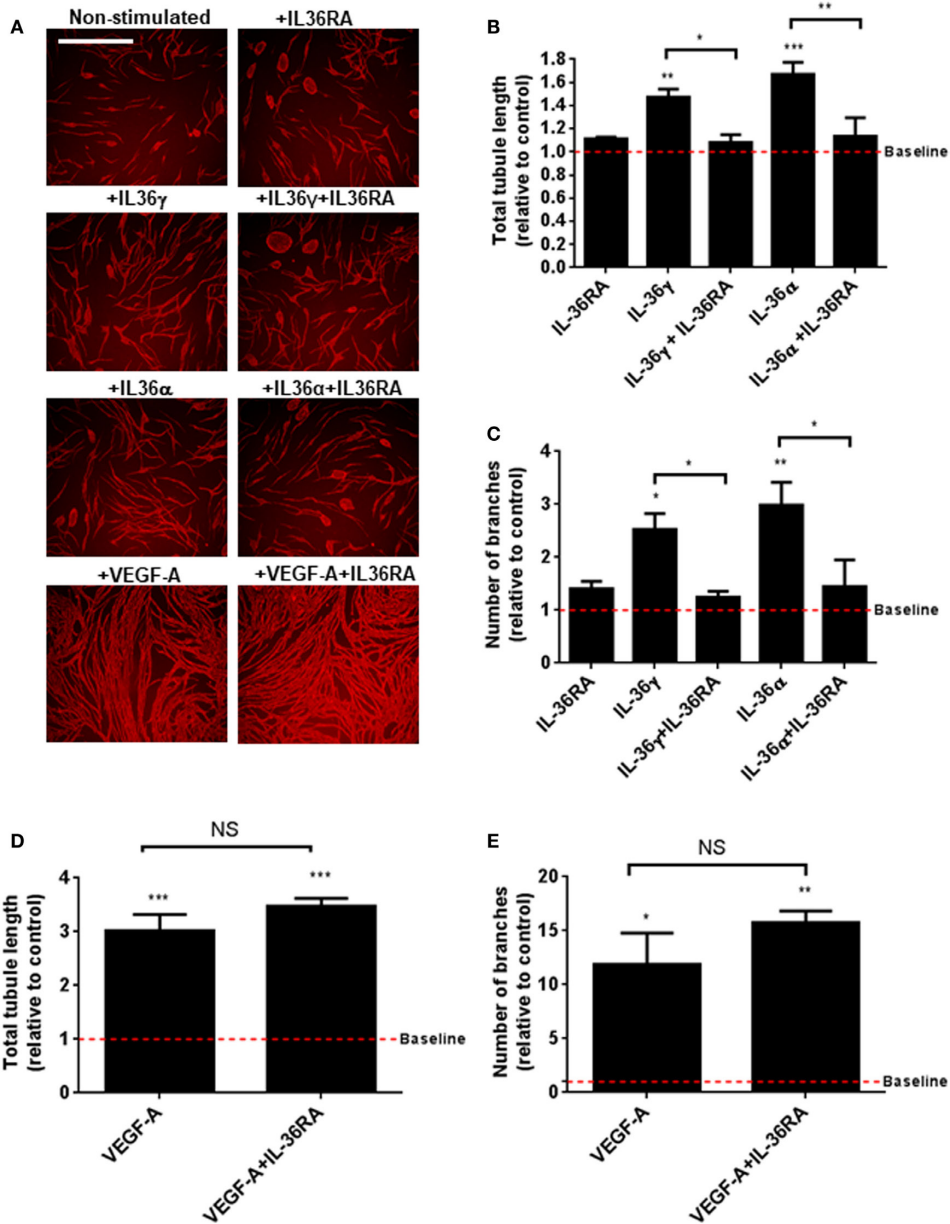
IL-36 stimulates endothelial cell tubulogenesis. **(A)** Human umbilical vein endothelial cells were cocultured on a bed of primary human fibroblasts for 9 days and stimulated with either IL-36 (50 ng/ml) or VEGF-A (10 ng/ml). Cocultures were then fixed and stained for PECAM-1, before visualisation using immunofluorescence microscopy. **(B,C)** Quantification of **(B)** total tubule length or **(C)** number of branches upon IL-36 stimulation. **(D,E)** Quantification of **(D)** total tubule length or **(E)** number of branches upon VEGF-A stimulation. Scale bar represents 1,000 µm (*n* = 3). One-way analysis of variance was performed (*p < 0.05, **p < 0.01, and ***p < 0.001).

IL-36’s relationship with angiogenesis in the context of inflammation is presently unknown. To close this knowledge gap, we investigated the role of IL-36 in blood vessel formation. Endothelial cell tubulogenesis was assessed using an endothelial-fibroblast coculture assay. Here, human endothelial cells were cocultured on a monolayer of primary human fibroblasts, before IL-36 or VEGF-A (positive control) stimulation, fixation, and visualisation of PECAM-1 positive endothelial cells (Figure 2A). Quantification revealed that both IL-36γ and IL-36α (50 ng/ml) stimulation produced a significant increase in both tubule length (Figure 2B) and branch point number (Figure 2C). Such effects were dependent on IL-36/IL-36R interactions, as treatment with an IL-36RA impaired IL-36-stimulated tubulogenesis (Figures 2A–C). Endothelial cell tubulogenesis was also enhanced in response to VEGF-A (10 ng/ml; Figures 2A,D,E); however, as expected, this was unaffected by co-treatment with IL-36RA (Figures 2A,D,E). Thus, these data show that IL-36R-mediated signal transduction promotes endothelial cell tubulogenesis.

### IL-36-Stimulated Endothelial Cell Tubulogenesis Is VEGF-A Dependent

**Figure 3:**
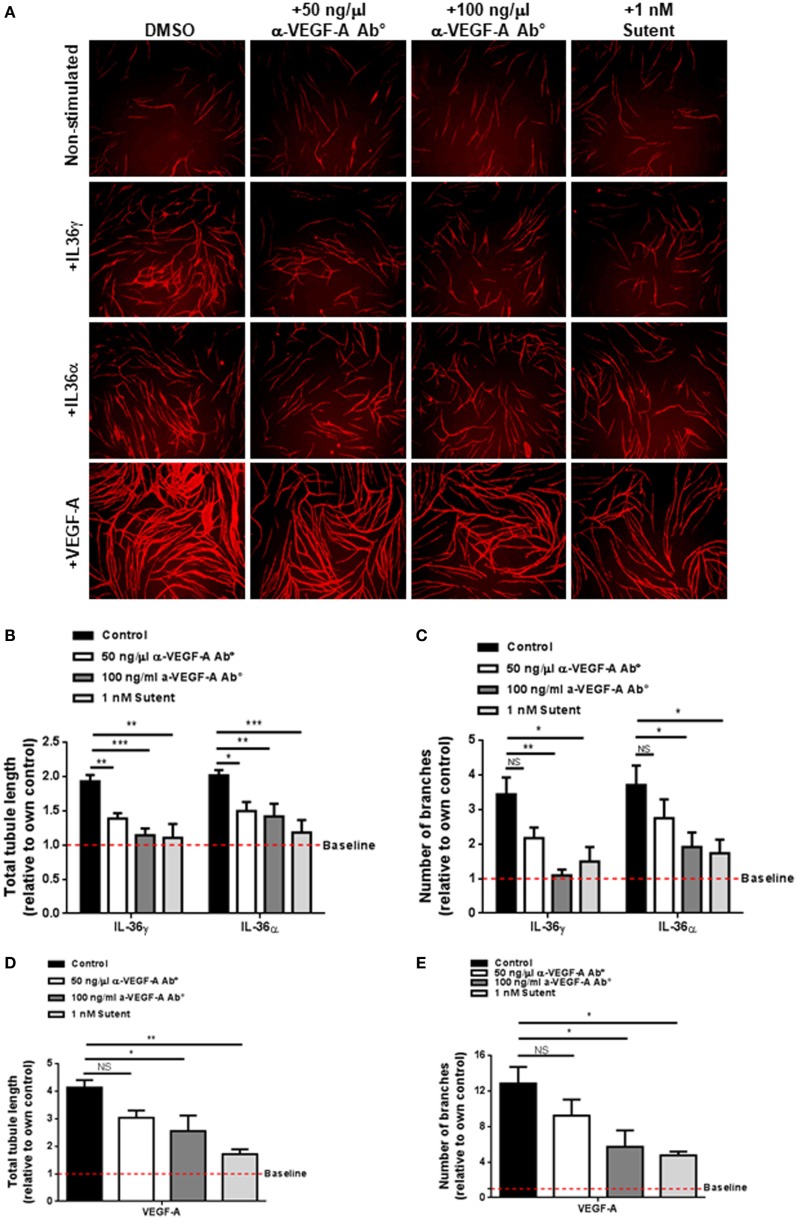
IL-36-stimulated endothelial cell tubulogenesis is VEGF-A dependent. **(A)** Human umbilical vein endothelial cells were cocultured on a bed of primary human fibroblasts for 9 days and stimulated with either IL-36 (50 ng/ml) or VEGF-A (10 ng/ml) ± 50 ng/ml anti-VEGF-A antibody, 100 ng/ml anti-VEGF-A antibody, or 1 nM Sutent. Cocultures were then fixed and stained for PECAM-1, before visualisation using immunofluorescence microscopy. **(B,C)** Quantification of **(B)** total tubule length or **(C)** number of branches upon IL-36 stimulation. **(D,E)** Quantification of **(D)** total tubule length or **(E)** number of branches upon VEGF-A stimulation. Scale bar represents 1,000 µm (*n* = 3). Two-way analysis of variance (ANOVA) **(C,D)** and one-way ANOVA **(D,E)** were applied (*p < 0.05, **p < 0.01, and ***p < 0.001).

VEGF-A is a strong promoter of angiogenesis (53) and endothelial cell tube formation (Figure 2). Pro-angiogenic molecules such as IL-6 have been shown to induce the expression and secretion of VEGF-A; therefore, one possibility was that IL-36-mediated endothelial cell tube formation could be dependent on increased VEGF-A signalling. To test this, human endothelial cells were cocultured on a bed of primary human fibroblasts and stimulated with IL-36 or VEGF-A (positive control) in the presence or absence of an anti-VEGF-A neutralising antibody or the VEGFR inhibitor, Sutent (Figure 3A). Here, quantification revealed that IL-36-stimulated (50 ng/ml) endothelial cell tubulogenesis was significantly impaired in response to either the anti-VEGF-A neutralising antibody (50 and 100 ng/µl) or Sutent (1 nM; Figures 3A–C). As expected, VEGF-A-stimulated endothelial cell tubulogeneis was also impaired in response to either the anti-VEGF-A neutralising antibody or Sutent (Figures 3A,D,E). Therefore, these data show that IL-36-stimulated endothelial cell tube formation is dependent on VEGF-A-mediated signal transduction.

### IL-36 Stimulation Upregulates VEGF-A Protein Levels in Fibroblast Cells

**Figure 4:**
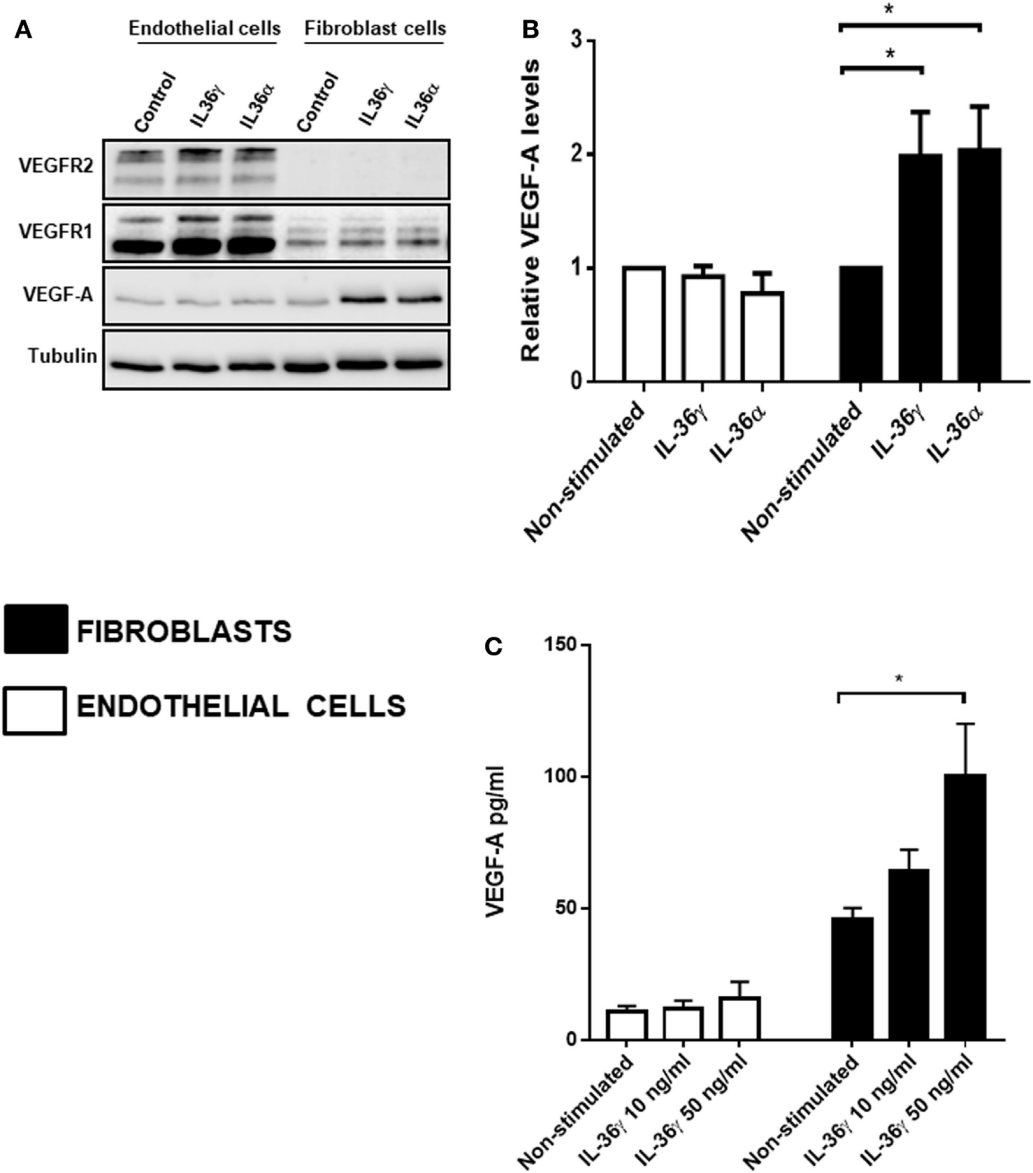
IL-36 stimulates VEGF-A expression and secretion by primary human fibroblasts. **(A)** Human umbilical vein endothelial cells or foreskin-derived fibroblasts were stimulated with IL-36 (50 ng/ml) for 24 h, before cell lysis. Endothelial cell or fibroblast lysates were processed for detection of VEGF-A protein levels *via* immunoblot analysis. **(B)** Quantification of VEGF-A protein levels upon IL-36 stimulation by 2D-densitometry (*n* = 3). **(C)** The supernatant from both the stimulated cell types was also tested for VEGF-A protein concentration. Two-way analysis of variance was performed (*p < 0.05).

After concluding IL-36-induced angiogenesis is dependent on VEGF-A-mediated signal transduction (Figure 3), we assessed the effect of IL-36 stimulation on the protein levels of VEGF-A and its receptors VEGFR1 and VEGFR2. Here, endothelial or fibroblast cells were serum-starved before IL-36 stimulation (50 ng/ml; 24 h); cells were then lysed and subjected to immunoblotting (Figure 3A). Here, IL-36 stimulation significantly increased VEGF-A protein levels (~2-fold) in primary fibroblasts (Figures 4A,B), but not in endothelial cells (Figures 4A,B). IL-36 also induced significant VEGF secretion by fibroblasts, but not endothelial cells, as detected by ELISA (Figure 4C). However, IL-36 stimulation had no significant effect on VEGFR1 or VEGFR2 protein levels in either cell type (Figure 4A). These data suggest that IL-36-induced VEGF-A secretion from surrounding fibroblasts cell is capable of stimulating endothelial cell tubulogenesis.

### Psoriasis Monocytes Show Increased Adhesion

**Figure 5:**
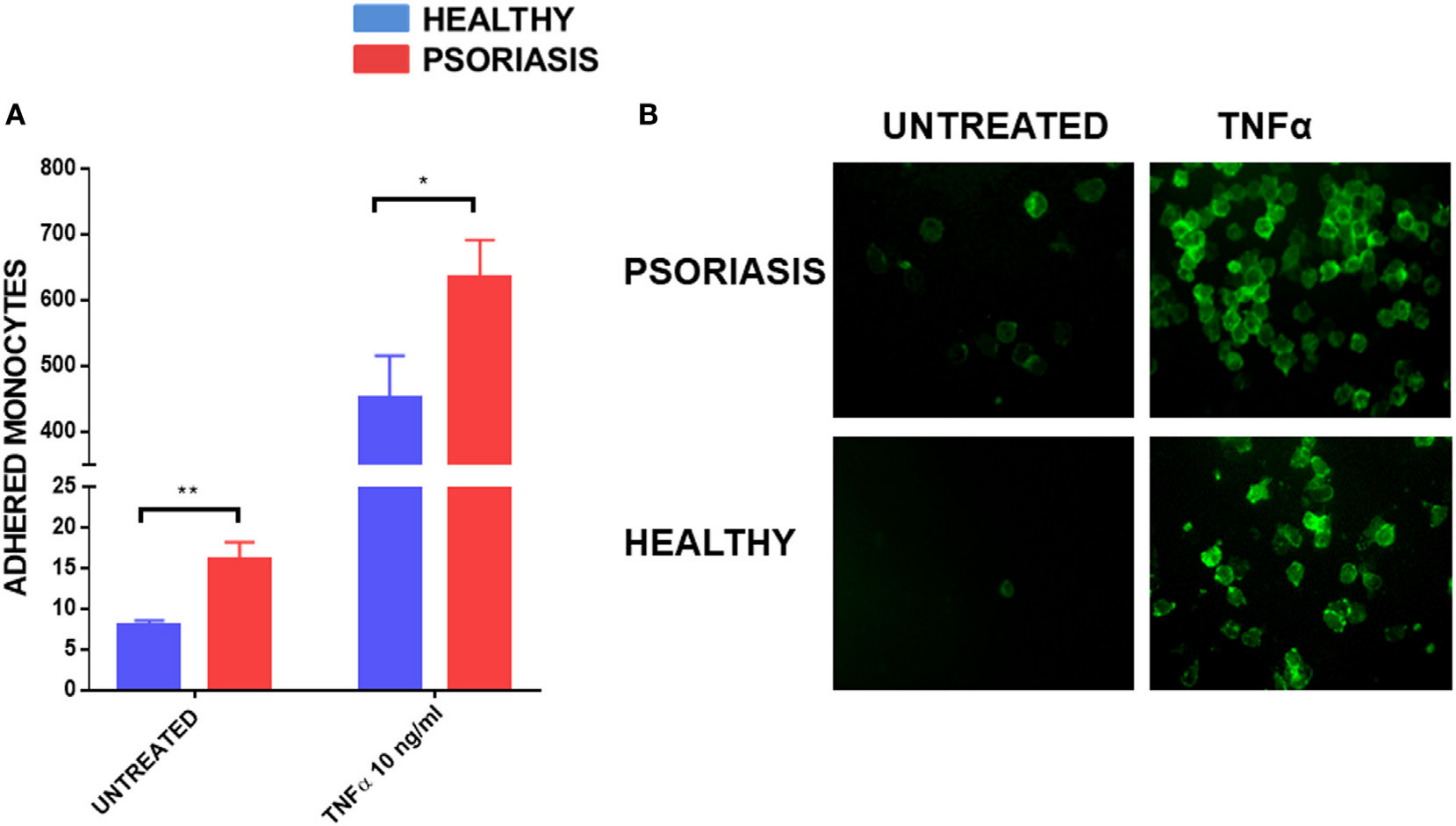
Human umbilical vein endothelial cell monolayer was stimulated with or without TNFα 10 ng for 24 h. 1 × 10^5^ monocytes were allowed to adhere to the monolayer for 30 min. Cells were visualised by immunofluorescence microscopy following CD14+ staining **(B)** and counted **(A)** (patient monocytes: psoriasis = 8; healthy = 8). Magnification 40×. Unpaired *t*-test *p < 0.05 and **p < 0.01.

As monocyte recruitment is crucial for psoriasis lesion development, but also comorbidities associated with psoriasis such as atherosclerosis, we decided to investigate the adhesive properties of psoriasis patients’ monocytes to HUVECs. To fully visualise potential differences in monocyte adhesion we worked with non-stimulated HUVEC monolayers but also used the best described stimulus, TNFα, to reliably upregulate adhesion molecules on endothelial cells (54). Psoriasis and healthy monocytes (healthy *n* = 8, psoriasis *n* = 8) were allowed to adhere for 30 min to HUVECs. Psoriasis patients’ monocytes showed increased adherence to both untreated and TNFα activated HUVECs (Figures 5A,B).

### IL-36γ-Stimulated Macrophage Supernatant Activates Endothelial Cells

**Figure 6:**
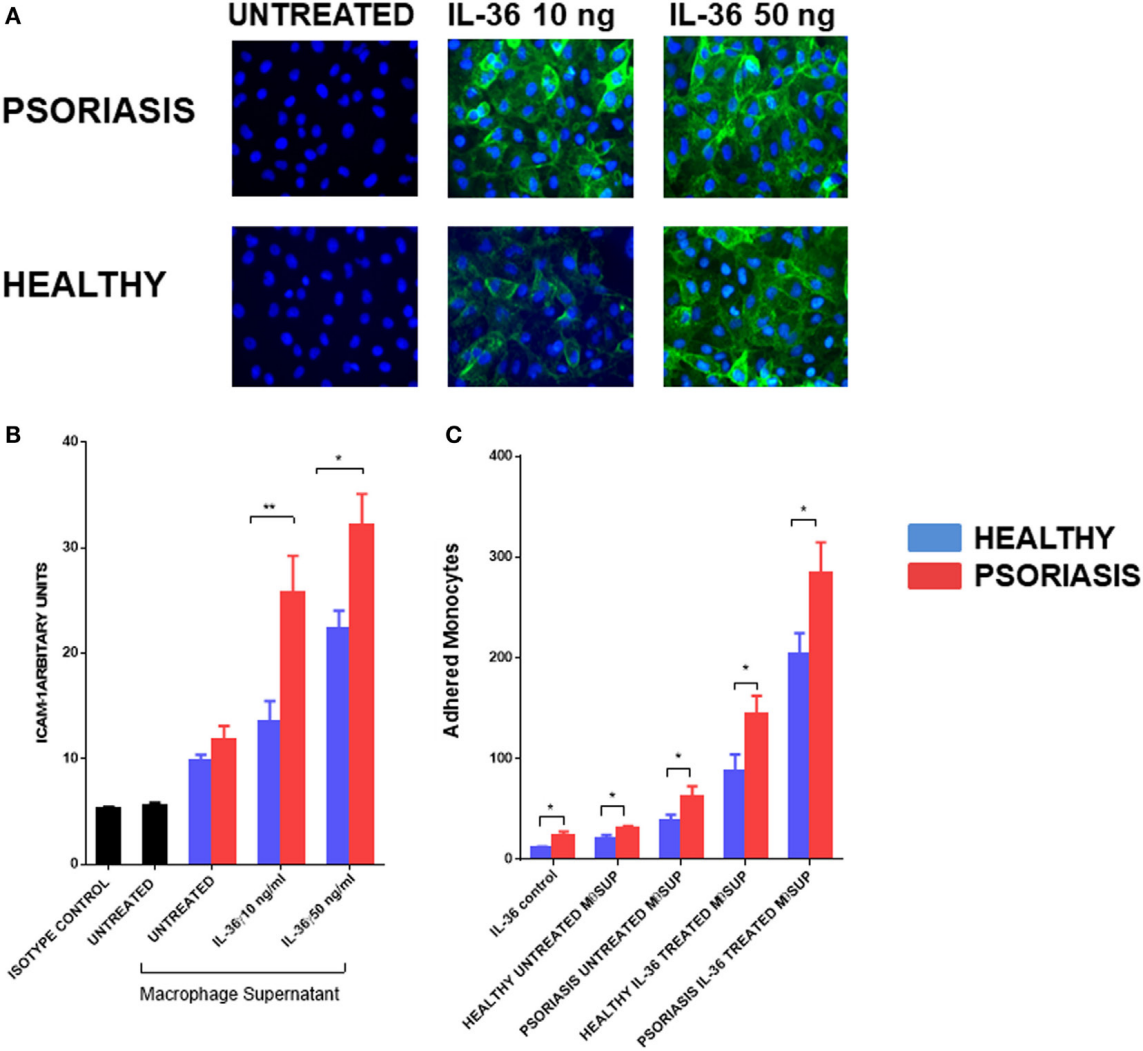
**(A,B)** Supernatants from IL-36γ-stimulated psoriasis or healthy macrophages were used to stimulate human umbilical vein endothelial cell (HUVECs) for 24 h, and ICAM-1 (green) and DAPI (blue) expression as visualised by immunofluorescence microscopy is depicted in panel **(A)** and intensity of staining measured by fluorescence absorbance summarised in panel [**(B)**; psoriasis *n* = 8, healthy *n* = 8]. **(C)** Healthy and psoriasis monocytes (1 × 10^5^) were allowed to adhere to a HUVEC monolayer for 30 min. The monolayer was stimulated with supernatant derived from IL-36-stimulated/non-stimulated psoriasis or healthy derived macrophage supernatant. Patient monocytes: psoriasis *n* = 8, healthy *n* = 8. Unpaired *t*-test *p < 0.05 and **p < 0.01.

To further understand the functional role of IL-36γ beyond the epidermal compartment we examined the aspect of immune cell recruitment into the skin using endothelial cells and monocytes/macrophages. Supernatants from IL-36γ-stimulated macrophages were incubated with endothelial cells (HUVECs) for 24 h at a ratio of 1:10. As IL-36 alone has pro-inflammatory effects on HUVEC, a recombinant control was also added. IL-36γ-stimulated supernatant markedly increased expression of the adhesion molecule ICAM-1 (Figures 6A,B). Supernatant derived from IL-36γ-stimulated, psoriasis macrophages induced significantly more ICAM-1 expression when compared with healthy macrophages (Figures 6A,B). Stimulation of HUVECs with psoriasis macrophage supernatant resulted in increased adhesion of both healthy and psoriasis monocytes (Figure 6C) (healthy *n* = 8, psoriasis *n* = 8). However, regardless of HUVEC stimulation (untreated or treated, supernatant from healthy or psoriasis macrophages), psoriasis monocytes showed increased endothelial adhesion.

## Discussion

IL-36γ is an IL-1 family cytokine with an increasingly recognised importance in the pathology of psoriasis (5). Various myeloid cells are thought to contribute to the pathology of psoriasis, including macrophages (33). Myeloid cells are capable of secreting IL-23 and thus contributing to the IL-23/IL-17 axis, prominent in psoriasis (36). The central role of a type-3 immune response shift in psoriatic inflammation is convincingly demonstrated by the overwhelmingly positive therapeutic response of biologics interfering with the IL-23/IL-17 axis, which leads to complete or almost complete clearing of psoriatic symptoms in a large number of patients receiving these treatments. Within psoriasis lesions, monocytes, macrophages and dendritic cells all show positive staining for IL-23 (55).

With IL-36γ being released by keratinocytes, probably in the context of cellular stress/environmental challenges, its downstream actions on dermal cells including fibroblasts, endothelial cells but also resident and infiltrating myeloid cells such as macrophages could represent a key step in both early and chronic lesion pathology. While IL-36γ has previously been shown to induce IL-23 mRNA in murine bone marrow derived dendritic cells, we here report secretion of IL-23 protein by human macrophages (56). Interestingly, other inflammatory cytokines prominent in psoriatic lesions, TNFα, IL-17, and IL-1, had little or no ability to induce IL-23 when compared with IL-36γ. Psoriasis macrophages secreted significantly more IL-23 following IL-36γ stimulation than healthy macrophages. Our findings also complement findings from an imiquimod-induced mouse model of psoriasis which has shown to be dependent on MyD88 signalling in macrophages (57). Whilst macrophage derived IL-23 is thought to be crucial to the immunopathological development of psoriasis lesions, we are the first to report a viable cytokine agonist for this induction, in IL-36γ. Clinical case reports also support our findings and the idea of a potential IL-36-macrophage pathway within psoriasis pathology. Two case reports show patients with DITRA, who suffer from a lack of function mutation in the endogenous IL-36RA, benefit from monocyte apheresis treatment (44, 58).

IL-36γ induced secretion of IL-23 was enhanced when macrophages were primed and activated with IFNγ. IFNγ enhancement of/priming for IL-23 secretion from macrophages has previously also been shown with other TIR agonists such as TLRs (50, 59). IFNγ has also been shown to induce an inflammatory phenotype characteristic of psoriasis when injected into the skin and serum levels correlate with disease severity (60, 61). In this context it is noteworthy that IL-36, similar to other IL-1 family members has been shown to enhance IFNγ production in CD4+ T cells (62, 63). We found in our experimental setting that synergy with IFNγ is a prominent feature for IL-36 induced responses but not for IL-17, TNFα, or the IL-1 family member and TIR agonist IL-1. Previous reports have also shown that IL-1 induced TNFα secretion from macrophages is not enhanced by IFNγ (64).

TNFα was prominently induced in macrophages by IL-36γ. Similar to IL-23, psoriasis macrophages had higher basal expression levels and secreted significantly more TNFα when stimulated with IL-36γ. Consequently, IL-36γ induced TNFα from infiltrating macrophages would be well placed to potently stimulate the surrounding tissues to further orchestrate the immune response, activate the endothelium, and increase leucocyte migration.

TNFα is known to be a prominent inflammatory activator of the endothelium and we show that IL-36 induced macrophage supernatant is a potent activator of the endothelium, with the adhesion molecule ICAM-1 showing upregulation (65). In accordance with enhanced cytokine secretion seen from psoriasis macrophages, their supernatant was able to achieve increased endothelial activation when compared with healthy supernatant. Deciphering the most important activator of the endothelium within the supernatant will require further study. Whilst, TNFα is a known activator of the endothelium and several biologic treatments targeting TNFα have proved successful in psoriasis conditions (66), IL-36γ is known to induce other cytokines from macrophages (including a positive autocrine feedback on its own production) which could induce similar effects. The key information from this set of experiments is the striking difference in the endothelium activating potential of IL-36-stimulated macrophages between healthy and psoriasis individuals. IL-36 is expressed in high abundance in all lesional psoriasis epidermis (5).

Enhanced cytokine secretion from psoriasis macrophages is characteristic of the exaggerated immune response associated with psoriasis (67). Previous studies have also shown psoriasis macrophages to secrete increased IL-8, IL-1α/β, and TNFα when untreated and this is in agreement with our findings (68, 69). Numerous genetic variations are thought to exist in psoriasis cells which lead to dysregulated immune responses (67). IL-36γ signals through NF-κB, and various variants—including TNFAIP3 (A20) and CARD14—are thought to exist within psoriasis cells that lead to increased activity of NF-κB (67, 70). Recent studies also suggest IL-36 may play a role in macrophage polarisation (40), and thereby affect subsequent cytokine secretion. However, whether IL-36 has a direct or indirect role in macrophage polarisation within psoriasis is as yet unknown.

Whilst damaged keratinocytes may be a potential source of IL-36γ, macrophages within lesions also show positive IL-36 staining (6). Interestingly, lung macrophages secrete IL-36γ in microparticles following LPS stimulation (71). Potential autocrine actions of IL-36 on macrophages thus require further study.

We report that following stimulation of endothelial cells with IL-36 induced macrophage supernatant, monocytes show significantly increased adherence. When compared with healthy controls, supernatants from psoriasis macrophages have an increased ability to stimulate and adhere to the endothelium (ICAM-1). Interestingly, regardless of the stimulus, psoriasis monocytes showed increased adhesion to both unstimulated and stimulated endothelial cells. A previous study has also found monocytes from psoriasis patients to show increased activation and integrin expression (72).

We report here that IL-36γ induced angiogenesis is dependent on VEGF-A induction. VEGF-A is perhaps the best documented inducer of angiogenesis, and its presence in psoriasis lesions is long established (73). VEGF-A and both VEGFRs are overexpressed in psoriasis lesions and serum levels of VEGF-A correlate with PASI (74–76). A genetic variant in *VEGFA* is also associated with more severe psoriasis (+405 CC) and is thought to result in increased VEGF-A production (29, 31). Interestingly, the same SNP is also associated with poor prognosis in patients with chronic heart failure (77). Importantly, it is thought that angiogenesis precedes symptomatic lesion formation; so it could be hypothesised that IL-36γ released from damaged keratinocytes would be well placed to stimulate VEGF-A synthesis and thus angiogenesis when compared with other cytokines that would have importance in a chronic lesion (78).

Angiogenesis has even been muted as a potential therapeutic target for psoriasis (20, 79, 80). Case reports have demonstrated improvements in PASI through targeting pro-angiogenic factors such as VEGF-A. Bevacizumab, a monoclonal antibody against VEGF-A used in the treatment for solid cancers, has also been shown to be effective in treating psoriasis, including one case of complete remission for a patient being treated for metastatic colon cancer (81). Case reports for tyrosine kinase inhibitors that target VEGFRs such as sunitinib and sorafenib also have produced positive results regarding psoriasis symptom reduction (82–84). Of interest, G6-31, a murine antibody against VEGF-A has demonstrated therapeutic improvement, in a mouse model of psoriasis (85). VALPHA is a fusion protein that targets both TNFα and VEGF-A and has shown to be effective in treating TPA induced psoriasis in mouse models (86).

The findings presented here also may have implications for other inflammatory diseases. Crohn’s shares some immunological aspects with psoriasis, namely the IL-23/IL-17 axis activation, and in addition, a potential role for IL-36 in Crohn’s is becoming apparent (6, 87). Interestingly, angiogenesis is also a feature of Crohn’s (88). Similarly, IL-36 has been implicated in mouse models of respiratory infection and again linked to the IL-23/IL-17 axis, and furthermore, angiogenesis is associated with chronic lung inflammation (40, 89, 90). Whilst IL-36 is yet to be fully implicated in COPD, cigarette smoke, the causative agent of COPD, induces IL-36 from bronchial epithelial cells (91). COPD is heavily associated with Th17 cell driven inflammation (92). Psoriasis is emerging as a risk factor for COPD, and furthermore, mouse models of psoriasis show enhanced airway inflammation attributed to IL-23 signalling (93–95). For skin diseases there is emerging evidence for IL-36 to be upregulated in pathologies with neutrophil components (e.g., acne and hidradenitis suppurativa) and to some extent in all inflammatory diseases involving epidermal responses (96). Although, difficult to dissect the precise *in vivo* relevance of the IL-36-induced VEGF-A mediated angiogenesis, multiple observations point towards an important potential role. Angiogenesis does play a physiological important role in healing responses where IL-36 could have an important impact (97). In a mouse model of psoriasis, systemic anti-VEGF-A treatment has also reduced skin inflammation (85). However, VEGF-A is also induced by other skin inflammatory mediators such as TNFα, and the net effect of IL-36 remains to be shown in future *in vivo* studies.

Our data greatly support previous data suggesting a role for IL-36 in the pathology of psoriasis. IL-36 has been shown to be intimately involved in the epidermal changes characterising psoriatic lesions. This study provides further evidence of a direct relationship between the development of a Th17 psoriatic phenotype and IL-36. IL-36 acts on tissue infiltrating macrophage and actively promotes recruitment of monocytes which cumulatively amplify IL-23 expression, thus promoting polarisation of lymphocytes for increased IL-17/IL-22 expression. In addition, IL-36 directed angiogenesis is dependent on VEGF, a recognised precursor to the development of a psoriatic plaque. We therefore demonstrate a central, pivotal role of IL-36 in the development and propagation of psoriatic disease. This builds on current understanding of psoriasis pathogenesis and provides a further potential therapeutic target in managing disease. Given its potential role in establishing a psoriatic plaque this may offer an opportunity to affect the disease course through preventing a chronic disease signature being established. Thus, deciphering the exact significance of IL-36 in the psoriatic disease continuum remains an important issue for further translational and clinical studies.

## Ethics Statement

This study was approved by Yorkshire and the Humber—Leeds West Research Ethics Committee with written informed consent from all subjects (PDAR study: REC 16/YH/0086).

## Author Contributions

CB wrote manuscript and conducted macrophage experiments. GF performed angiogenesis experiments and help write the manuscript. AB, PL, and MW delivered the clinical ethical and patient-related aspects of the projects and obtained clinical samples. PL and SP contributed to critical appraisal of results. TM generated and provided IL-36 molecules. MS, MW, and AG contributed to experimental planning, critical discussion of results obtained, as well as manuscript correction.

## Conflict of Interest Statement

The authors declare that the research was conducted in the absence of any commercial or financial relationships that could be construed as a potential conflict of interest.

## Supplementary Material

Figure S2 Monocytes were isolated from PBMCs using magnetic separation CD14+ beads. CD14+ purity was tested by FACs analysis with mouse anti-human CD14 FITC conjugated or mouse IgG isotype control Purity for healthy and diseased patients was >90.

## References

[1] Nestle FO Kaplan DH Barker J Psoriasis. N Engl J Med (2009) 361(5):496–509. 10.1056/NEJMra0804595 19641206

[2] Lowes MA Suárez-Fariñas M Krueger JG. Immunology of psoriasis. Annu Rev Immunol (2014) 32:227–55. 10.1146/annurev-immunol-032713-120225 24655295 PMC4229247

[3] Towne JE Sims JE IL-36 in psoriasis. Curr Opin Pharmacol (2012) 12(4):486–90. 10.1016/j.coph.2012.02.009 22398321

[4] He Q Chen H.-x Li W Wu Y Chen S.-j Yue Q IL-36 cytokine expression and its relationship with p38 MAPK and NF-κB pathways in psoriasis vulgaris skin lesions. J Huazhong Univ Sci Technol Med Sci (2013) 33(4):594–9. 10.1007/s11596-013-1164-1 23904383

[5] D’Erme AM Wilsmann-Theis D Wagenpfeil J Holzel M Ferring-Schmitt S Sternberg S IL-36[gamma] (IL-1F9) is a biomarker for psoriasis skin lesions. J Invest Dermatol (2015) 135(4):1025–32. 10.1038/jid.2014.532 25525775

[6] Boutet MA Bart G Penhoat M Amiaud J Brulin B Charrier C Distinct expression of interleukin (IL)-36alpha, beta and gamma, their antagonist IL-36Ra and IL-38 in psoriasis, rheumatoid arthritis and Crohn’s disease. Clin Exp Immunol (2016) 184(2):159–73. 10.1111/cei.12761 26701127 PMC4837235

[7] Marrakchi S Guigue P Renshaw BR Puel A Pei X-Y Fraitag S Interleukin-36-receptor antagonist deficiency and generalized pustular psoriasis. N Engl J Med (2011) 365(7):620–8. 10.1056/NEJMoa1013068 21848462

[8] Kanazawa N Nakamura T Mikita N Furukawa F. Novel IL36RN mutation in a Japanese case of early onset generalized pustular psoriasis. J Dermatol (2013) 40(9):749–51. 10.1111/1346-8138.12227 23834760

[9] Sugiura K Takemoto A Yamaguchi M Takahashi H Shoda Y Mitsuma T The majority of generalized pustular psoriasis without psoriasis vulgaris is caused by deficiency of interleukin-36 receptor antagonist. J Invest Dermatol (2013) 133(11):2514–21. 10.1038/jid.2013.230 23698098

[10] Balato A Mattii M Caiazzo G Raimondo A Patruno C Balato N IL-36gamma is involved in psoriasis and allergic contact dermatitis. J Invest Dermatol (2016) 136(7):1520–3. 10.1016/j.jid.2016.03.020 27021407

[11] Di Caprio R Balato A Caiazzo G Lembo S Raimondo A Fabbrocini G IL-36 cytokines are increased in acne and hidradenitis suppurativa. Arch Dermatol Res (2017) 309(8):673–8. 10.1007/s00403-017-1769-5 28852851

[12] Carrier Y Ma HL Ramon HE Napierata L Small C O’Toole M Inter-regulation of Th17 cytokines and the IL-36 cytokines in vitro and in vivo: implications in psoriasis pathogenesis. J Invest Dermatol (2011) 131(12):2428–37. 10.1038/jid.2011.234 21881584

[13] Tortola L Rosenwald E Abel B Blumberg H Schafer M Coyle AJ Psoriasiform dermatitis is driven by IL-36-mediated DC-keratinocyte crosstalk. J Clin Invest (2012) 122(11):3965–76. 10.1172/jci63451 23064362 PMC3484446

[14] Medina-Contreras O Harusato A Nishio H Flannigan KL Ngo V Leoni G Cutting edge: IL-36 receptor promotes resolution of intestinal damage. J Immunol (2016) 196(1):34–8. 10.4049/jimmunol.1501312 26590314 PMC4684965

[15] Ainscough JS Macleod T McGonagle D Brakefield R Baron JM Alase A Cathepsin S is the major activator of the psoriasis-associated proinflammatory cytokine IL-36γ. Proc Natl Acad Sci U S A (2017) 114(13):E2748–57. 10.1073/pnas.1620954114 28289191 PMC5380102

[16] Foster AM Baliwag J Chen CS Guzman AM Stoll SW Gudjonsson JE IL-36 promotes myeloid cell infiltration, activation, and inflammatory activity in skin. J Immunol (2014) 192(12):6053–61. 10.4049/jimmunol.1301481 24829417 PMC4048788

[17] Li N Yamasaki K Saito R Fukushi-Takahashi S Shimada-Omori R Asano M Alarmin function of cathelicidin antimicrobial peptide LL37 through IL-36gamma induction in human epidermal keratinocytes. J Immunol (2014) 193(10):5140–8. 10.4049/jimmunol.1302574 25305315

[18] Bridgewood C Stacey M Alase A Lagos D Graham A Wittmann M IL-36gamma has proinflammatory effects on human endothelial cells. Exp Dermatol (2017) 26(5):402–8. 10.1111/exd.13228 27673278

[19] Scheibe K Backert I Wirtz S Hueber A Schett G Vieth M IL-36R signalling activates intestinal epithelial cells and fibroblasts and promotes mucosal healing in vivo. Gut (2017) 66(5):823–38. 10.1136/gutjnl-2015-310374 26783184

[20] Heidenreich R Röcken M Ghoreschi K. Angiogenesis drives psoriasis pathogenesis. Int J Exp Pathol (2009) 90(3):232–48. 10.1111/j.1365-2613.2009.00669.x 19563608 PMC2697548

[21] Braverman IM Yen A. Ultrastructure of the capillary loops in the dermal papillae of psoriasis. J Invest Dermatol (1977) 68(1):53–60. 10.1111/1523-1747.ep12485169 830771

[22] Braverman IM Sibley J. Role of the microcirculation in the treatment and pathogenesis of psoriasis. J Invest Dermatol (1982) 78(1):12–7. 10.1111/1523-1747.ep12497850 7054305

[23] Rosina P Giovannini A Gisondi P Girolomoni G. Microcirculatory modifications of psoriatic lesions during topical therapy. Skin Res Technol (2009) 15(2):135–8. 10.1111/j.1600-0846.2008.00336.x 19622121

[24] Kulka JP Microcirculatory impairment as a factor in inflammatory tissue damage. Ann N Y Acad Sci (1964) 116:1018–44. 10.1111/j.1749-6632.1964.tb52565.x 14212844

[25] Detmar M Brown LF Schon MP Elicker BM Velasco P Richard L Increased microvascular density and enhanced leukocyte rolling and adhesion in the skin of VEGF transgenic mice. J Invest Dermatol (1998) 111(1):1–6. 10.1046/j.1523-1747.1998.00262.x 9665379

[26] Xia YP Li B Hylton D Detmar M Yancopoulos GD Rudge JS. Transgenic delivery of VEGF to mouse skin leads to an inflammatory condition resembling human psoriasis. Blood (2003) 102(1):161–8. 10.1182/blood-2002-12-3793 12649136

[27] Trembath RC Clough RL Rosbotham JL Jones AB Camp RD Frodsham A Identification of a major susceptibility locus on chromosome 6p and evidence for further disease loci revealed by a two stage genome-wide search in psoriasis. Hum Mol Genet (1997) 6(5):813–20. 10.1093/hmg/6.5.813 9158158

[28] Brogan IJ Khan N Isaac K Hutchinson JA Pravica V Hutchinson IV. Novel polymorphisms in the promoter and 5’ UTR regions of the human vascular endothelial growth factor gene. Hum Immunol (1999) 60(12):1245–9. 10.1016/S0198-8859(99)00132-9 10626738

[29] Diaz BV Lenoir MC Ladoux A Frelin C Demarchez M Michel S. Regulation of vascular endothelial growth factor expression in human keratinocytes by retinoids. J Biol Chem (2000) 275(1):642–50. 10.1074/jbc.275.1.642 10617662

[30] Detmar M Evidence for vascular endothelial growth factor (VEGF) as a modifier gene in psoriasis. J Invest Dermatol (2004) 122(1):xiv–xv. 10.1046/j.0022-202X.2003.22140.x 14962120

[31] Young HS Summers AM Read IR Fairhurst DA Plant DJ Campalani E Interaction between genetic control of vascular endothelial growth factor production and retinoid responsiveness in psoriasis. J Invest Dermatol (2006) 126(2):453–9. 10.1038/sj.jid.5700096 16385345

[32] Puig L. The role of IL 23 in the treatment of psoriasis. Expert Rev Clin Immunol (2017) 13(6):525–34. 10.1080/1744666x.2017.1292137 28165883

[33] Clark RA Kupper TS. Misbehaving macrophages in the pathogenesis of psoriasis. J Clin Invest (2006) 116(8):2084–7. 10.1172/jci29441 16886055 PMC1523394

[34] Stratis A Pasparakis M Rupec RA Markur D Hartmann K Scharffetter-Kochanek K Pathogenic role for skin macrophages in a mouse model of keratinocyte-induced psoriasis-like skin inflammation. J Clin Invest (2006) 116(8):2094–104. 10.1172/jci27179 16886058 PMC1525004

[35] Wang H Peters T Kess D Sindrilaru A Oreshkova T Van Rooijen N Activated macrophages are essential in a murine model for T cell-mediated chronic psoriasiform skin inflammation. J Clin Invest (2006) 116(8):2105–14. 10.1172/JCI27180 16886059 PMC1523400

[36] Zaba LC Fuentes-Duculan J Eungdamrong NJ Abello MV Novitskaya I Pierson KC Psoriasis is characterized by accumulation of immunostimulatory and Th1/Th17 cell-polarizing myeloid dendritic cells. J Invest Dermatol (2009) 129(1):79–88. 10.1038/jid.2008.194 18633443 PMC2701224

[37] Fuentes-Duculan J Suarez-Farinas M Zaba LC Nograles KE Pierson KC Mitsui H A subpopulation of CD163-positive macrophages is classically activated in psoriasis. J Invest Dermatol (2010) 130(10):2412–22. 10.1038/jid.2010.165 20555352 PMC2939947

[38] Blumberg H Dinh H Dean C Jr Trueblood ES Bailey K Shows D IL-1RL2 and its ligands contribute to the cytokine network in psoriasis. J Immunol (2010) 185(7):4354–62. 10.4049/jimmunol.1000313 20833839

[39] Chi HH Hua KF Lin YC Chu CL Hsieh CY Hsu YJ IL-36 signaling facilitates activation of the NLRP3 inflammasome and IL-23/IL-17 axis in renal inflammation and fibrosis. J Am Soc Nephrol (2017) 28(7):2022–37. 10.1681/asn.2016080840 28179433 PMC5491282

[40] Kovach MA Singer B Martinez-Colon G Newstead MW Zeng X Mancuso P IL-36gamma is a crucial proximal component of protective type-1-mediated lung mucosal immunity in Gram-positive and -negative bacterial pneumonia. Mucosal Immunol (2017) 10(5):1320–34. 10.1038/mi.2016.130 28176791 PMC5548659

[41] Thomi R Kakeda M Yawalkar N Schlapbach C Hunger RE. Increased expression of the interleukin-36 cytokines in lesions of hidradenitis suppurativa. J Eur Acad Dermatol Venereol (2017) 31(12):2091–6. 10.1111/jdv.14389 28602023

[42] Zebrowska A Wozniacka A Juczynska K Ociepa K Waszczykowska E Szymczak I Correlation between IL36alpha and IL17 and activity of the disease in selected autoimmune blistering diseases. Mediators Inflamm (2017) 2017:8980534 10.1155/2017/8980534 28611508 PMC5458385

[43] Sugiura K Haruna K Suga Y Akiyama M Generalized pustular psoriasis caused by deficiency of interleukin-36 receptor antagonist successfully treated with granulocyte and monocyte adsorption apheresis. J Eur Acad Dermatol Venereol (2014) 28(12):1835–6. 10.1111/jdv.12383 24490830

[44] Koike Y Okubo M Kiyohara T Fukuchi R Sato Y Kuwatsuka S Granulocyte and monocyte apheresis can control juvenile generalized pustular psoriasis with mutation of IL36RN. Br J Dermatol (2017) 177(6):1732–6. 10.1111/bjd.15509 28369922

[45] Eccles KA Sowden H Porter KE Parkin SM Homer-Vanniasinkam S Graham AM. Simvastatin alters human endothelial cell adhesion molecule expression and inhibits leukocyte adhesion under flow. Atherosclerosis (2008) 200(1):69–79. 10.1016/j.atherosclerosis.2007.12.018 18486135

[46] Macleod T Doble R McGonagle D Wasson CW Alase A Stacey M Neutrophil elastase-mediated proteolysis activates the anti-inflammatory cytokine IL-36 receptor antagonist. Sci Rep (2016) 6:24880. 10.1038/srep24880 27101808 PMC4840362

[47] Fearnley GW Smith GA Odell AF Latham AM Wheatcroft SB Harrison MA Vascular endothelial growth factor A-stimulated signaling from endosomes in primary endothelial cells. Methods Enzymol (2014) 535:265–92. 10.1016/b978-0-12-397925-4.00016-x 24377929

[48] Fearnley GW Wheatcroft SB Ponnambalam S. Detection and quantification of vascular endothelial growth factor receptor tyrosine kinases in primary human endothelial cells. Methods Mol Biol (2015) 1332:49–65. 10.1007/978-1-4939-2917-7_4 26285745

[49] Hao JQ. Targeting interleukin-22 in psoriasis. Inflammation (2014) 37(1):94–9. 10.1007/s10753-013-9715-y 23978911

[50] van de Wetering D de Paus RA van Dissel JT van de Vosse E Salmonella induced IL-23 and IL-1β allow for IL-12 production by monocytes and Mϕ1 through induction of IFN-γ in CD56(+) NK/NK-like T cells. PLoS One (2009) 4(12):e8396 10.1371/journal.pone.0008396 20027291 PMC2791865

[51] Peral de Castro C Jones SA Ni Cheallaigh C Hearnden CA Williams L Winter J Autophagy regulates IL-23 secretion and innate T cell responses through effects on IL-1 secretion. J Immunol (2012) 189(8):4144–53. 10.4049/jimmunol.1201946 22972933

[52] Dietrich D Martin P Flacher V Sun Y Jarrossay D Brembilla N Interleukin-36 potently stimulates human M2 macrophages, Langerhans cells and keratinocytes to produce pro-inflammatory cytokines. Cytokine (2016) 84:88–98. 10.1016/j.cyto.2016.05.012 27259168

[53] Hoeben A Landuyt B Highley MS Wildiers H Van Oosterom AT De Bruijn EA. Vascular endothelial growth factor and angiogenesis. Pharmacol Rev (2004) 56(4):549–80. 10.1124/pr.56.4.3 15602010

[54] Mattila P Majuri ML Mattila PS Renkonen R. TNF alpha-induced expression of endothelial adhesion molecules, ICAM-1 and VCAM-1, is linked to protein kinase C activation. Scand J Immunol (1992) 36(2):159–65. 10.1111/j.1365-3083.1992.tb03087.x 1380176

[55] Yawalkar N Tscharner GG Hunger RE Hassan AS. Increased expression of IL-12p70 and IL-23 by multiple dendritic cell and macrophage subsets in plaque psoriasis. J Dermatol Sci (2009) 54(2):99–105. 10.1016/j.jdermsci.2009.01.003 19264456

[56] Vigne S Palmer G Lamacchia C Martin P Talabot-Ayer D Rodriguez E IL-36R ligands are potent regulators of dendritic and T cells. Blood (2011) 118(22):5813–23. 10.1182/blood-2011-05-356873 21860022

[57] Costa S Marini O Bevilacqua D DeFranco AL Hou B Lonardi S Role of MyD88 signaling in the imiquimod-induced mouse model of psoriasis: focus on innate myeloid cells. J Leukoc Biol (2017) 102(3):791–803. 10.1189/jlb.3MA0217-054RR 28642279 PMC6608051

[58] Tominaga C Yamamoto M Imai Y Yamanishi K. A case of old age-onset generalized pustular psoriasis with a deficiency of IL-36RN (DITRA) treated by granulocyte and monocyte apheresis. Case Rep Dermatol (2015) 7(1):29–35. 10.1159/000380876 25848350 PMC4357681

[59] Butchar JP Parsa KVL Marsh CB Tridandapani S IFNγ enhances IL-23 production during *Francisella* infection of human monocytes. FEBS Lett (2008) 582(7):1044–8. 10.1016/j.febslet.2008.02.058 18319062 PMC2376054

[60] Abdallah MA Abdel-Hamid MF Kotb AM Mabrouk EA. Serum interferon-gamma is a psoriasis severity and prognostic marker. Cutis (2009) 84(3):163–8. 19842576

[61] Johnson-Huang LM Suárez-Fariñas M Pierson KC Fuentes-Duculan J Cueto I Lentini T A single intradermal injection of IFN-γ induces an inflammatory state in both non-lesional psoriatic and healthy skin. J Invest Dermatol (2012) 132(4):1177–87. 10.1038/jid.2011.458 22277938 PMC3305841

[62] Renne J Schäfer V Werfel T Wittmann M. Interleukin-1 from epithelial cells fosters T cell-dependent skin inflammation. Br J Dermatol (2010) 162(6):1198–205. 10.1111/j.1365-2133.2010.09662.x 20128791

[63] Vigne S Palmer G Martin P Lamacchia C Strebel D Rodriguez E IL-36 signaling amplifies Th1 responses by enhancing proliferation and Th1 polarization of naive CD4+ T cells. Blood (2012) 120(17):3478–87. 10.1182/blood-2012-06-439026 22968459

[64] Ikejima T Okusawa S Ghezzi P van der Meer JW Dinarello CA. Interleukin-1 induces tumor necrosis factor (TNF) in human peripheral blood mononuclear cells in vitro and a circulating TNF-like activity in rabbits. J Infect Dis (1990) 162(1):215–23. 10.1093/infdis/162.1.215 2113076

[65] Meekins JW McLaughlin PJ West DC McFadyen IR Johnson PM. Endothelial cell activation by tumour necrosis factor-alpha (TNF-alpha) and the development of pre-eclampsia. Clin Exp Immunol (1994) 98(1):110–4. 10.1111/j.1365-2249.1994.tb06615.x 7523006 PMC1534159

[66] Yost J Gudjonsson JE. The role of TNF inhibitors in psoriasis therapy: new implications for associated comorbidities. F1000 Med Rep (2009) 1:30. 10.3410/M1-30 20948750 PMC2924720

[67] Harden JL Krueger JG Bowcock AM. The immunogenetics of psoriasis: a comprehensive review. J Autoimmun (2015) 64:66–73. 10.1016/j.jaut.2015.07.008 26215033 PMC4628849

[68] Okubo Y Koga M. Peripheral blood monocytes in psoriatic patients overproduce cytokines. J Dermatol Sci (1998) 17(3):223–32. 10.1016/S0923-1811(98)00019-X 9697051

[69] Nishibu A Han GW Iwatsuki K Matsui T Inoue M Akiba H Overexpression of monocyte-derived cytokines in active psoriasis: a relation to coexistent arthropathy. J Dermatol Sci (1999) 21(1):63–70. 10.1016/S0923-1811(99)00031-6 10468194

[70] Sun Y Mozaffarian A Arnett HA Dinh H Trueblood ES Towne JE 253. Cytokine (2013) 63(3):303 10.1016/j.cyto.2013.06.256

[71] Kovach MA Singer BH Newstead MW Zeng X Moore TA White ES IL-36gamma is secreted in microparticles and exosomes by lung macrophages in response to bacteria and bacterial components. J Leukoc Biol (2016) 100(2):413–21. 10.1189/jlb.4A0315-087R 26864267 PMC4945350

[72] Golden JB Groft SG Squeri MV Debanne SM Ward NL McCormick TS Chronic psoriatic skin inflammation leads to increased monocyte adhesion and aggregation. J Immunol (2015) 195(5):2006–18. 10.4049/jimmunol.1402307 26223654 PMC4686256

[73] Marina ME Roman II Constantin A-M Mihu CM TĂTaru AD. VEGF involvement in psoriasis. Clujul Med (2015) 88(3):247–52. 10.15386/cjmed-494 26609252 PMC4632878

[74] Detmar M Brown LF Claffey KP Yeo KT Kocher O Jackman RW Overexpression of vascular permeability factor/vascular endothelial growth factor and its receptors in psoriasis. J Exp Med (1994) 180(3):1141–6. 10.1084/jem.180.3.1141 8064230 PMC2191647

[75] Young HS Summers AM Bhushan M Brenchley PE Griffiths CE. Single-nucleotide polymorphisms of vascular endothelial growth factor in psoriasis of early onset. J Invest Dermatol (2004) 122(1):209–15. 10.1046/j.0022-202X.2003.22107.x 14962110

[76] Nofal A Al-Makhzangy I Attwa E Nassar A Abdalmoati A. Vascular endothelial growth factor in psoriasis: an indicator of disease severity and control. J Eur Acad Dermatol Venereol (2009) 23(7):803–6. 10.1111/j.1468-3083.2009.03181.x 19309427

[77] van der Meer P de Boer RA White HL van der Steege G Hall AS Voors AA The VEGF +405 CC promoter polymorphism is associated with an impaired prognosis in patients with chronic heart failure: a MERIT-HF substudy. J Card Fail (2005) 11(4):279–84. 10.1016/j.cardfail.2004.11.006 15880336

[78] Telner P Fekete Z The capillary responses in psoriatic skin. J Invest Dermatol (1961) 36:225–30. 10.1038/jid.1961.36 13775806

[79] Guérard S Pouliot R The role of angiogenesis in the pathogenesis of psoriasis: mechanisms and clinical implications. J Clin Exp Dermatol Res (2012) S2:7 10.4172/2155-9554.S2-007

[80] Weidemann AK Crawshaw AA Byrne E Young HS. Vascular endothelial growth factor inhibitors: investigational therapies for the treatment of psoriasis. Clin Cosmet Investig Dermatol (2013) 6:233–44. 10.2147/CCID.S35312 24101875 PMC3790838

[81] Akman A Yilmaz E Mutlu H Ozdogan M. Complete remission of psoriasis following bevacizumab therapy for colon cancer. Clin Exp Dermatol (2009) 34(5):e202–4. 10.1111/j.1365-2230.2008.02991.x 19077094

[82] Fournier C Tisman G. Sorafenib-associated remission of psoriasis in hypernephroma: case report. Dermatol Online J (2010) 16(2):17. 20178713

[83] Narayanan S Callis-Duffin K Batten J Agarwal N. Improvement of psoriasis during sunitinib therapy for renal cell carcinoma. Am J Med Sci (2010) 339(6):580–1. 10.1097/MAJ.0b013e3181dd1aa5 20421784

[84] Antoniou EA Koutsounas I Damaskos C Koutsounas S. Remission of psoriasis in a patient with hepatocellular carcinoma treated with sorafenib. In Vivo (2016) 30(5):677–80. 27566090

[85] Schonthaler HB Huggenberger R Wculek SK Detmar M Wagner EF. Systemic anti-VEGF treatment strongly reduces skin inflammation in a mouse model of psoriasis. Proc Natl Acad Sci U S A (2009) 106(50):21264–9. 10.1073/pnas.0907550106 19995970 PMC2795522

[86] Jung K Lee D Lim HS Lee SI Kim YJ Lee GM Double anti-angiogenic and anti-inflammatory protein Valpha targeting VEGF-A and TNF-alpha in retinopathy and psoriasis. J Biol Chem (2011) 286(16):14410–8. 10.1074/jbc.M111.228130 21345791 PMC3077640

[87] Siakavellas SI Bamias G. Role of the IL-23/IL-17 axis in Crohn’s disease. Discov Med (2012) 14(77):253–62. 23114581

[88] Alkim C Alkim H Koksal AR Boga S Sen I. Angiogenesis in inflammatory bowel disease. Int J Inflam (2015) 2015:970890. 10.1155/2015/970890 26839731 PMC4709626

[89] Matarese A Santulli G. Angiogenesis in chronic obstructive pulmonary disease: a translational appraisal. Transl Med UniSa (2012) 3:49–56. 23905052 PMC3728789

[90] Kovach MA Newstead MW Zeng X Peters-Golden M Standiford TJ IL-36γ is a potent inducer of type I and IL-17 cytokine induction during lung infection. Am J Respir Care Med (2015) 191: A6148.

[91] Parsanejad R Fields WR Steichen TJ Bombick BR Doolittle DJ. Distinct regulatory profiles of interleukins and chemokines in response to cigarette smoke condensate in normal human bronchial epithelial (NHBE) cells. J Interferon Cytokine Res (2008) 28(12):703–12. 10.1089/jir.2008.0139 18937544

[92] Alcorn JF Crowe CR Kolls JK. TH17 cells in asthma and COPD. Annu Rev Physiol (2010) 72:495–516. 10.1146/annurev-physiol-021909-135926 20148686

[93] Dreiher J Weitzman D Shapiro J Davidovici B Cohen AD. Psoriasis and chronic obstructive pulmonary disease: a case-control study. Br J Dermatol (2008) 159(4):956–60. 10.1111/j.1365-2133.2008.08749.x 18637897

[94] Li X Kong L Li F Chen C Xu R Wang H Association between psoriasis and chronic obstructive pulmonary disease: a systematic review and meta-analysis. PLoS One (2015) 10(12):e0145221. 10.1371/journal.pone.0145221 26700640 PMC4689442

[95] Nadeem A Al-Harbi NO Ansari MA Al-Harbi MM El-Sherbeeny AM Zoheir KM Psoriatic inflammation enhances allergic airway inflammation through IL-23/STAT3 signaling in a murine model. Biochem Pharmacol (2017) 124:69–82. 10.1016/j.bcp.2016.10.012 27984001

[96] Walsh PT Fallon PG. The emergence of the IL-36 cytokine family as novel targets for inflammatory diseases. Ann N Y Acad Sci (2016). 10.1111/nyas.13280 27783881

[97] Tonnesen MG Feng X Clark RA Angiogenesis in wound healing. J Investig Dermatol Symp Proc (2000) 5(1):40–6. 10.1046/j.1087-0024.2000.00014.x 11147674

