# Supplementary material for: IL-36γ Is a Strong Inducer of IL-23 in Psoriatic Cells and Activates Angiogenesis
Source: Front Immunol. 2018 Feb 26;9:200. doi: 10.3389/fimmu.2018.00200 (PMC5834930; doi:10.3389/fimmu.2018.00200)
Supplement: Supplementary file 1 [file image_1.PDF]

Additional Figure 1

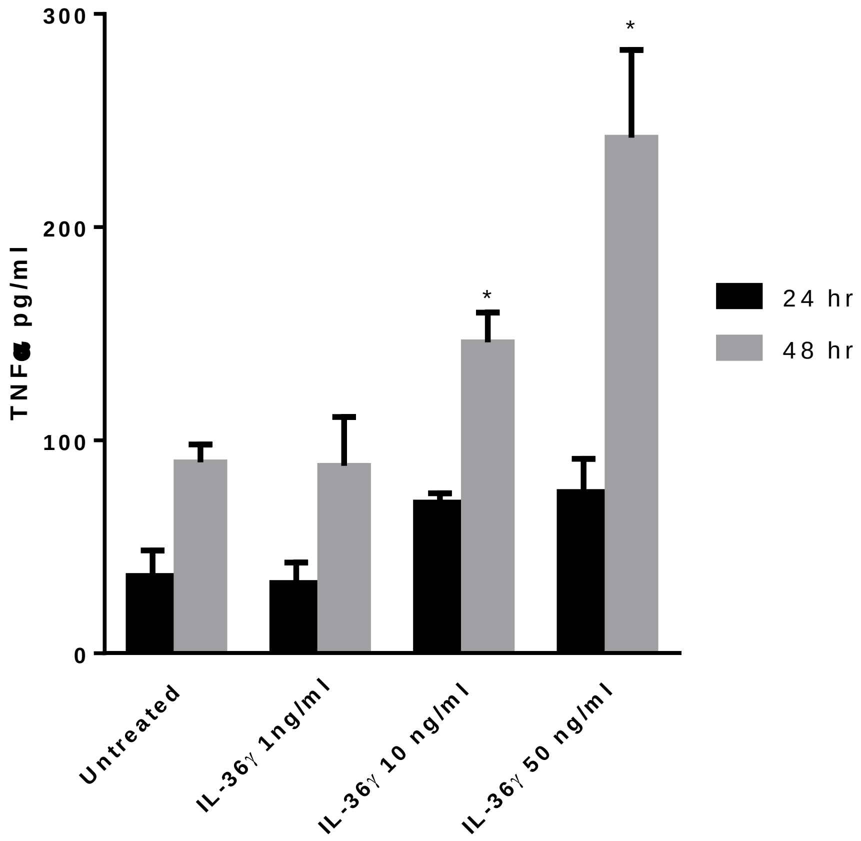

Figure 1. Healthy macrophages express TNFα significantly at 48 hr post IL-36 stimulation. Macrophages were isolated as described in text, and stimulated with either IL-36γ (1, 10 and 100 ng). TNFα secretion was measured by ELISA into the supernatant at both 24 and 48 hr. Unpaired t-test *= p<0.05 from untreated control. (n=3).
