# Supplementary figures and images for: IL-36γ Is a Strong Inducer of IL-23 in Psoriatic Cells and Activates Angiogenesis
Source: Front Immunol. 2018 Feb 26;9:200. doi: 10.3389/fimmu.2018.00200 (PMC5834930; doi:10.3389/fimmu.2018.00200)

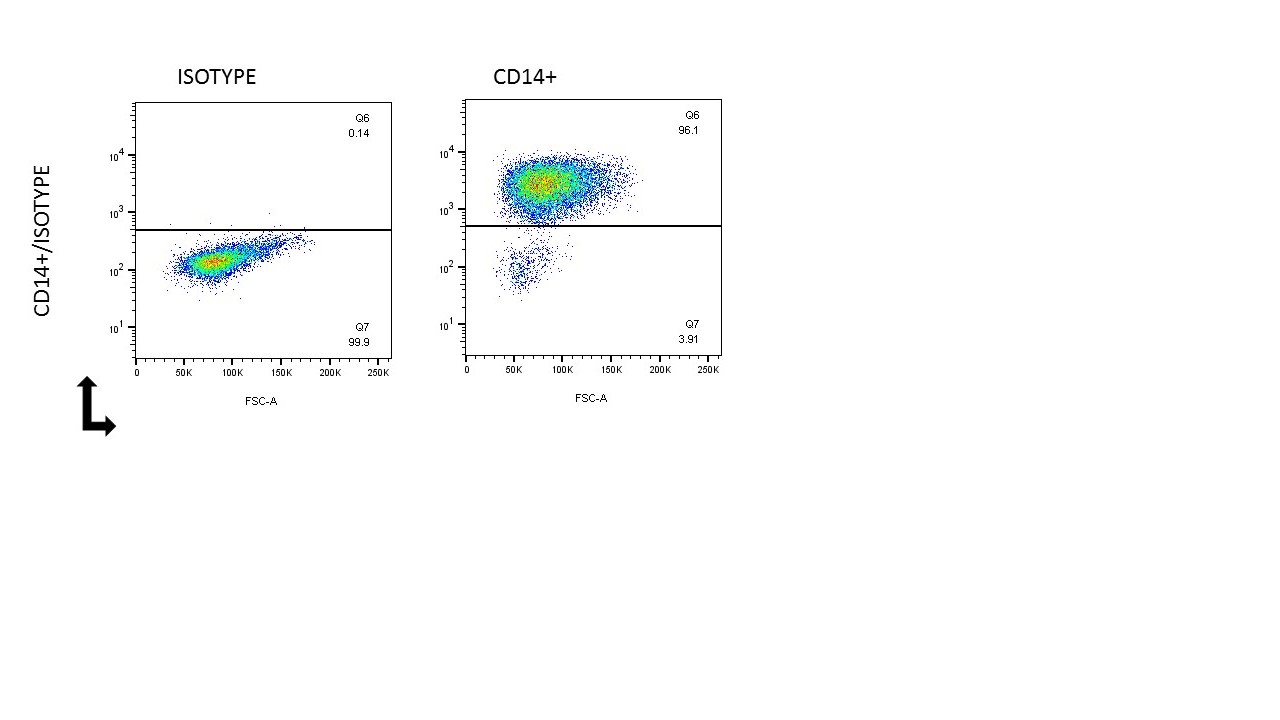

Supplement: Figure S2 — Monocytes were isolated from PBMCs using magnetic separation CD14+ beads. CD14+ purity was tested by FACs analysis with mouse anti-human CD14 FITC conjugated or mouse IgG isotype control Purity for healthy and diseased patients was >90. [file image_2.jpeg]
